# The Missing Quality of Tuberculosis Care and Treatment Delivered in Public-Health Facilities, Northeast Ethiopia: A Cross-Sectional Study

**DOI:** 10.3390/clinpract12060106

**Published:** 2022-12-12

**Authors:** Asrat Agalu Abejew, Hussen Abdu, Yimer Seid, Agumas Shibabaw

**Affiliations:** 1 Department of Pharmacy, College of Medicine and Health Sciences, Bahir Dar University, Bahir Dar P.O. Box 79, Ethiopia; 2 School of Medicine, College of Medicine and Health Sciences, Wollo University, Dessie P.O. Box 1145, Ethiopia; 3 Department of Pharmacy, College of Medicine and Health Sciences, Wollo University, Dessie P.O. Box 1145, Ethiopia; 4 Department of Medical Laboratory Sciences, Medical Microbiology Unit, College of Medicine and Health Sciences, Wollo University, Dessie P.O. Box 1145, Ethiopia

**Keywords:** tuberculosis, treatment outcome, quality of care, health facilities, Ethiopia

## Abstract

Tuberculosis (TB) remains a major global public-health problem. TB prevention and control measures are compromised by poor quality of care delivered to TB patients in health facilities during diagnosis, treatment, and follow-up; thus, this study was intended to determine the quality of TB care and treatment delivered in public-health facilities in Northeast Ethiopia. A cross-sectional study was conducted in health facilities in South Wollo zone from January to April 2018. Data were collected from each study participant through face-to-face interviews. A TB registration logbook was reviewed for every registered TB patient and compiled using a structured questionnaire and standard checklists. The quality of care for each health facility was graded as very good, good, marginal, poor, and very poor if health facilities achieved [90–100%], [80–90%), [70–80%), [60–70%), and <60% of performance indicators, respectively, using the Donabedian structure, process, and outcome model of healthcare quality. All the health facilities had at least one functional microscope, and all the facilities had sufficient TB drugs almost all the time. All the facilities had reported to have sufficient laboratory reagents and slides for sputum smear microscopy. Of 1579 patients registered, 18.5% and 66.1% were cured and successfully completed the course of treatment, respectively. The overall quality of TB care and treatment was good (72.5%), and ranged from 70.9% to 74.8% among health facilities. Outcome (83.4%) and process (80%) qualities of care were very good but the structural quality of care was very poor. In conclusion, the overall quality of TB care and treatment analysed in this study was found to be good. There should be an integrated approach to improve the quality of TB care and treatment in health facilities in Ethiopia. Based on the findings, continuous supply of anti-TB drugs, laboratory equipment and reagents, availing current guidelines, providing up to-date training for healthcare workers, and proper documentation are important to improve the quality of care delivered to TB patients.

## 1. Introduction

Tuberculosis (TB) is a major global public-health problem. Ethiopia is among the 10 countries with the highest triple burden (TB, TB/HIV, and MDR-TB) in the world [1]. The current End TB strategy begins with early diagnosis, treatment of all TB patients, and combined TB/HIV focus. This imposes high demands on resource provision which are extremely difficult to fulfill in countries with under-developed economies. Delivering good-quality care for TB patients is crucial for the prevention and control of the disease, and reduces morbidity and mortality due to TB [1].

Several factors affect the accessibility and effectiveness of TB care and treatment adherence in developing countries, including Ethiopia [2,3]. First, structural factors related to organization and management in health facilities used to assess the extent to which TB care is accessible and convenient to patients include the presence of a health worker trained in TB care, a separate TB treatment unit, provision of daily outpatient TB care, uninterrupted TB drugs supply, and patient treatment monitoring [2,3]. Second, process factors constitute ingredients of TB care delivered to patients by TB-focused healthcare workers, while treatment outcomes are regarded as outcomes of structural and/or process factors [2,3]. The quality of patient care delivered by a TB-focused healthcare worker depends on the level of WHO-recommended structural inputs implemented by health facilities and district TB control programs [4]. 

Decentralizing TB care from tertiary to primary healthcare facilities has had a major positive impact on Ethiopia’s TB treatment outcomes [4,5]. Most countries of Sub-Saharan Africa have failed to achieve the global target for treatment success rate. The directly observed therapy short-course (DOTS) strategy also demands effective management of the TB control program, including continuous supplies, training, and supervision of health providers, and program monitoring and evaluation [6]. 

In handling patients who have TB or who are suspected of having TB, all practitioners should strive to provide treatment that meets the standard outlined in the international standards for TB care (ISTC). The standards are designed to make it easier for all healthcare professionals to work together effectively to offer high-quality care for patients of all ages, including those who have TB caused by drug-resistant *Mycobacterium tuberculosis*, extra-pulmonary TB, and TB–HIV co-infection [7].

A positive relationship or interaction between patients and healthcare workers (HCWs) may lead to good treatment outcomes and vice versa. The success of the relationship is affected by the knowledge of the HCWs about the disease and treatment protocol, their skills on counseling and educating patients, and their attitudes towards the patients. Patients who are poorly educated or counseled about TB and its treatment may end up with poor outcomes; similarly, a negative attitude of the HCWs towards patients may cause them to stop the treatment [2,8].

Ethiopia continues to experience considerable morbidity and mortality from tuberculosis despite the use of various preventive and control strategies; the poor quality of care and treatment given to TB patients in various health facilities (HFs) is one of the contributing reasons [2,9,10]. In many resource-limited nations, including Ethiopia, access to high-quality healthcare is a top goal for public health [11]; however, studies are scarce on the quality of TB care and treatment delivered in public-health facilities in South Wollo zone, Northeast Ethiopia; thus, the present study was conducted to determine the quality of TB care and treatment delivered to TB patients in the study area. 

## 2. Materials and Methods 

### 2.1. Study Design and Settings 

A facility based cross-sectional study was conducted from January to April 2018 in Dessie and Kombolcha town health facilities, South Wollo zone. A total of seven and three health facilities were found in Dessie and Kombolcha town, respectively (eight primary, one secondary, and one tertiary-level health facilities). Two hospitals (Dessie Referral Hospital and Boru Meda General Hospital) and two urban health centers (Dessie Health Center and Kombolcha Health Center) were selected from all the health facilities in the two towns based on experience in TB care and treatment. The DOTS program is implemented in each health facility based on the national TB program guidelines. Smear microscopy, clinical examination, X-ray, and GeneXpert assays were used for diagnosis of tuberculosis. Smear microscopy is the common method for diagnosing and monitoring treatment in all health facilities in Ethiopia, which does not ensure the detection of drug-resistant TB and the presence of HIV infection which necessitates appropriate treatment correction. Continuous professional development trainings are provided for every health professional working in TB care, treatment, and diagnosis (in-service training on TB guidelines, DOTS, diagnostic tests, quality controls and errors, etc.), and regular supervision and monitoring are conducted.

### 2.2. Study Participants

Dessie Referral Hospital, Boru Meda General Hospital, Dessie Health Center, and Kombolcha Health Center participated in the study. All TB patients who completed their treatment were included in the retrospective cohort, and those visiting the TB DOTS clinic during the study period were included in the study. We included the TB patients who were registered from December 2014 to December 2017 in order to determine the TB treatment outcomes, and performance of the health professionals assigned at the TB DOTS clinic. From each health facility, two TB-focused people were included and interviewed to assess the knowledge of the service providers about TB care. 

### 2.3. Population and Inclusion Criteria 

A study population were randomly selected public HFs and healthcare workers (HCWs) working in the TB DOTS clinic at public HFs of Dessie and Kombolcha town. All TB patients registered for three consecutive years in either HF and all HCWs who worked at least for the past three months in her/his current health facility’s TB DOTS clinic were included in the study. Any healthcare workers who were on break leave or who had less than three months’ experience were excluded from the study. 

### 2.4. Data-Collection Process

All HFs were using similar TB registration logbooks. Data were collected using questionnaires and checklists to capture data on the quality of TB care and treatment based on structure, process, and outcome. The instruments used to assess structure, process, and outcome dimensions were developed and customized based on the Ethiopian National TB Laboratory guidelines [4].

Checklists were used to assess the structural attributes of quality of TB care based on five categories: infrastructure, access, management and staffing, drugs and diagnostic testing, and patient environment. 

The Donabedian structure, process, and outcome model was used to assess the performance of the health professionals assigned at the TB clinic, and to evaluate process and outcome quality of TB care and treatment [12]. An average score for structural quality was computed by assigning a score of 1 for the presence of that aspect or positive responses and a score of 0 for the absence of that aspect or negative responses of each aspect. The percentage score for each health facility was calculated, and structural quality was classified as: very good [90–100%], good [80–90%), marginal [70–80%), poor [60–70%), and very poor (<60%) [12].

The process quality was measured using a structured interview guide to assess the knowledge of health professionals who were involved in the management of TB patients and/or TB-focused people to measure the performance of the health professionals who were working in the TB DOTS clinic during the study period. The health professionals’ knowledge was assessed using eight structured questions. Process qualities related to patients were collected by interviewing the patients at the completion of their treatment in the health facilities. 

The outcome quality was evaluated based on the registered TB patients through tracing their patient cards at each health facility, and assessing the success and loss to follow-up rate at each health facility. The patient registration logbook was used to determine the treatment outcome and composite index for overall quality of TB care. To assess quality of registration, items were audited from the unit’s TB register: patient name, age, sex, weight, address of patient, start date of intensive phase, 1–4 sputum smear microscopy, category of treatment, type of TB, drugs administered during intensive and continuation phase, and treatment outcome were all extracted from TB logbook. Quality of unit TB registration was assessed by assigning a score of 1 for recorded items and 0 for unrecorded items. 

The percentage score for each record was calculated and quality of medical recording was classified as: very good [90–100%], good [80–90%), marginal [70–80%), poor [60–70%), and very poor (<60%) [12].

### 2.5. Operational Definitions

**Structural quality:** The structural aspects of quality of care delivered at each health facility and overall health facilities’ level (infrastructure, access, management and staffing, drugs and diagnostic testing, and patient environment) were assessed using 7, 11, 10, 5, and 6 questions/items, respectively. 

**Process quality:** The process aspects of healthcare quality mainly included TB providers, availability of laboratory and pharmacy units, and patient TB knowledge. Eight questions were designed to assess the knowledge of healthcare workers on TB causative agent, transmission, treatment, treatment-monitoring mechanisms, prevention, and control methods. By considering the total maximum attainable score as 100%, the actual score was summed and percentage was calculated and the quality of TB care provided by health facilities was graded based on the percentages achieved. The following questions/items were included to assess the availability of equipment such as microscopes, laboratory materials, reagents, TB guidelines, anti-TB drugs, and training of health professionals. By considering the total maximum attainable score as 100%, the actual score was summed and percentage was calculated and the quality of care provided by HFs was graded based on the percentages achieved. Patient TB knowledge, patients–HCWs interaction, and understanding of services were assessed using 15 items/questions.

**Outcome quality:** The outcome quality aspects of care were assessed using the data collected from a three-year document review of registered TB patients using a checklist containing 12 items including TB patient-treatment outcomes; then considering the total maximum attainable score as 100%, the actual score was summed and percentage was calculated and the quality of care provided by HFs was graded based on the percentage achieved.

### 2.6. Data Analysis 

Data were entered, cleaned, and analyzed using SPSS version 20 software. Descriptive statistics were employed to determine median, percentage, and relative frequencies, and the results were presented using tables. 

## 3. Results

A total of 1579 patient data were abstracted from the TB record logbook. All the HFs had at least one functional microscope and in all HFs there were sufficient TB drugs for almost all the time. All the HFs had reported to have sufficient laboratory reagents and slides for sputum smear microscopy; however, drugs were stored on the table during the observation period. None of the HFs were involved in surveillance for TB, annual radiological screening, or contact-tracing except self-reporting of cases at health centers reported for contact-tracing by health extension workers. TB diagnosis was mainly conducted by sputum smear microscopy at all health facilities.

### 3.1. Demographic and Clinical Characteristics of Patients

At all health facilities, the smear results were not regularly recorded and, likewise, the duration of treatment for all intensive and continuous phases of therapy were not recorded completely. At all health facilities, sex, TB type, treatment category, and treatment outcomes were almost completely recorded in the registration logbook. 

Of 1579 registered TB patients in the record, the median age of the patient was 30 (IQR, 18) years. The majority of patients, 924 (58.5%) and 751 (47.6%) were males and in the 15–29 years age group, respectively. New TB cases accounted for 1454 (92.1%) of the cases recorded in the logbook. The majority of patients with TB (66.1%) completed their treatment, and lost-to-follow-up and failure cases accounted for 33 (2.1%) and 25 (1.6%), respectively (Table 1).

### 3.2. Structural Quality of TB Care and Treatment 

The overall structural quality was graded as very poor at all health facilities. It was poor in the areas of patient environment, infrastructure, management, and staffing when compared with other parameters but relatively better in access, drugs, and diagnostic facilities (Table 2). 

### 3.3. Process Quality of TB Care and Treatment 

The overall process quality of TB care and treatment was very good (80%) for all the health facilities. A total of eight TB-focused health professionals participated in the TB care knowledge assessment. Eight TB-focused professionals had very good (100%) knowledge about the cardinal symptoms of TB, anti-TB drug categories, and side-effects of anti-TB drugs but despite their knowledge not all TB-focused professionals managed minor illness or counselled patients on treatment, due to workload. 

Forty (40) patients (10 patients at each HF) who had completed their treatment at the four health facilities were interviewed. More than half the patients (55%) knew that bacteria caused TB and 32.5% of patients did not. All patients (100%) knew it was curable and had a good knowledge of its signs and symptoms. Thirty one patients (77.5%) were literate and had completed their education from primary school through to college. No patient encountered a problem when seeking care and there was no variation in the provision of care to TB patients.

### 3.4. Outcome Quality of TB Care and Treatment

The overall outcome quality was very good (82.6%) at the health facilities. The overall quality of treatment outcome considering those who were cured and who completed their treatment was marginal (65.5%), while it was very poor at Dessie’s comprehensive specialized hospital (34%) and very good at Dessie Health Center (89.9%) (Table 3).

### 3.5. Overall Quality of TB Care and Treatment 

The average score for quality of TB care and treatment at each health facility was good with the score of 70–73. The overall quality of TB care and treatment at all health facilities is rated as 0.725 (72.5%) which is the average score of all health facilities. The overall quality of TB care and treatment was good (Table 4).

## 4. Discussion

The treatment and prevention of TB should be customized to the patients’ health needs. Improvements in patient adherence to TB protocols are likely due to patient-centered care at healthcare facilities and good management support from healthcare providers. Our study assessed the quality of TB care and treatment delivered at health facilities to support implementers, service providers, and all those bodies concerned with improving the quality of care provided to TB patients to reduce the disease toll in the country. 

Similar to findings by Jimma [10], our study found that all the HFs evaluated had a very poor structural quality of TB care and treatment (53.6%). Our study found this lower than was found by the studies conducted in North Shewa zone (60%) and Sidama zone (85%) [2,13]. This might be due to variations of resource mobilization, program managers’ and health institution leaders’ attention, and infrastructure limitation. The present study also indicated that all HFs had sufficient anti-TB drugs supply. This finding was in agreement with studies in Uganda [14] and Ethiopia (Debre Tabor [9], Jimma [10], and Bahir Dar [15]); moreover, all HFs had at least one functional microscope used for TB diagnostic service which was similar with studies by Jimma and Debre Tabor [9,10]; on the other hand, there were no separate toilet facilities and this fact was not comparable with studies by Debre Tabor and Jimma [9,10]. 

Moreover, the process quality of TB care was very good (80%), which was comparable with the study by Jimma [10] for assessing the knowledge of TB among TB-focused health workers. TB-focused workers had very good knowledge (100%) about the cardinal symptoms of TB, anti-TB drug categories, and side-effects of TB drugs as compared to a study by Tigray, which accounted for 39% in managing drug side-effects [6]. Not all TB-focused professionals managed minor illness or counselled patients on treatment at all levels due to workload and there was a difference in the type of questions that mostly concerned them.

Patients had very good (55%) general knowledge about the causative agent of TB in this study as compared to the study by Tigray [6], which accounted for 12%. This might be due to variation in sample size, health facility settings, and the fact that most patients were literate in our finding and easily understood the correct causative agent and transmission methods of TB. No patients encountered a problem when seeking care and there was no variation in provision of care between patients. This might be due to the present HFs being dedicated to creating awareness and providing continuous education for the community through health extension workers.

In the present study, all TB patients’ TB registration number, age, and sex were properly recorded, in agreement with a study by Bahir Dar and Debre Tabor, Ethiopia [9,15]. The present study showed that the outcome quality was graded as very good (83.4%) which was higher than in the study documented by Jimma (42%) [10]. The treatment outcomes rating was 68.8%, which was comparable with the same study (69%) [10]. A majority of TB cases completed (66.1%) their treatment, which was comparable with the study by Tigray (65.4%), but the lost-to-follow-up rate was low (2.1%) in the present study as compared to a similar study, which accounted for 22% [6]. This might be due to variation in providing patient health education regarding treatment adherence, side-effects of anti-TB drugs, and implications of being lost to follow-up. 

The present study revealed that not all HFs had the new version of the national TB guidelines, which agrees with the studies by Jimma and Debre Tabor [10,15]. The low finding observed in this study might be explained by the fact that not all HFs had the new version of the TB manual [4] referred to in the national TB guidelines.

Furthermore, the overall quality of TB care and treatment was good (72.5%) as compared to the study by Jimma [10]. This might be due to variation in the healthcare settings, study area, and criteria for measuring the quality of TB care and treatment. 

The good quality of care in TB services helps patients and their families to address their health needs safely and effectively. Ensuring provision of good-quality TB care at health facilities is an important component of TB control strategy to reduce poor medical practices which result in drug-resistant TB. The national TB program should emphasise participatory in-service training for HCWs, focusing on patient-centered continuous quality improvement, teamwork, and implementation of infection prevention and control practices. To enhance the quality of TB service, we recommend to assess and improve the quality of TB services at all levels for all health facilities in Ethiopia through intervention, guidance, continuous monitoring, and evaluation strategy.

The limitation of this study might be that the quality of TB diagnosis was only verified by what was written in the patient record logbook. Due to this present study’s retrospective character, certain variables were overlooked or there were incomplete records. The fact that just the TB-focused worker was interviewed and that only a small number of patients participated raises the possibility of bias in the assessment of the process quality.

## 5. Conclusions

The overall standard of care and treatment for TB was found to be good. This study showed the need for numerous improvements, including in the areas of management and staffing, patient environment, and structural quality aspects, especially infrastructure (adequate toilets, working refrigerator, staff incentives, emergency kits, and medication storage). All health facilities ought to enhance the way smear results are recorded. It is necessary to construct a system to trace patients who are lost to follow-up. Strict adherence to all the DOTS strategy components could guarantee the effectiveness of anti-TB treatments.

## Figures and Tables

**Table 1:** Demographic and clinical characteristics of patients from the TB DOTS clinic logbook (N = 1579) at public-health facilities, Northeast Ethiopia, from January to April 2018.

Characteristics		Frequency n (%)
Age (years)	<15	118 (7.5)
	15–29	751 (47.6)
	30–45	561 (35.5)
	>45	149 (9.4)
Sex	Male	924 (58.5)
	Female	655 (41.5)
Site of TB infection	Pulmonary TB	985 (62.4)
	Extra-pulmonary TB	591 (37.4)
	Both	3 (0.2)
History of TB	New cases	1454 (92.1)
	Previously treated cases	125 (7.9)
Treatment outcome	Cured	293 (18.6)
	Completed	1043 (66.1)
	Died	101(6.4)
	Failed	25 (1.6)
	Lost to follow-up	33 (2.1)
	Transferred out	84 (5.3)

**Table 2:** Structural quality score of public-health facilities providing TB care at public-health facilities in Northeast Ethiopia from January to April 2018.

Health Facility	Infrastructure	Access	Management and Staffing	Drugs and Diagnostic Testing	Patient Environment	Average Score No (%)	Quality
DRH	3/7	8/11	6/10	4/5	0/6	21 (55.3)	Very poor
BMH	2/7	8/11	6/10	4/5	0/6	20 (52.6)	Very poor
KHC	2/7	8/11	7/10	4/5	0/6	21 (55.3)	Very poor
DHC	1/7	8/11	7/10	4/5	0/6	20 (52.6)	Very poor
	Average score					(53.95)	Very poor

DRH: Dessie Referral Hospital; BMH: Boru Meda Hospital; KHC: Kombolcha Health Center; and DHC: Dessie Health Center.

**Table 3:** Performance of healthcare workers in TB care and treatment delivery at public-health facilities in Northeast Ethiopia from January to April 2018.

Characters	DRH	BMH	DHC	KHC	Average Score
Patient name recorded	100	100	100	100	100
Age recorded	100	98.1	100	98	99.025
Sex recorded	100	94.3	100	97	97.825
Weight recorded	100	98.1	100	99.5	99.4
Address recorded	100	97.2	100	100	99.3
Start date recorded	0	0	0	0	0
1–4 sputum smear microscopy	3.8	0	0	0	0.95
Category of treatment	100	90.6	100	97	96.9
Type of TB recorded	100	84.9	100	100	96.225
Drug administered during intensive phase	97.1	94.3	99.7	98	97.275
Drug administered during continuation phase	17.1	67.9	90.5	58.5	58.5
Treatment outcome/success	34	67.9	89.9	70	63.35
Average score	76.7	81.6	89.1	83.5	82.6

DRH: Dessie Referral Hospital; BMH: Boru Meda Hospital; DHC: Dessie Health Center; and KHC: Kombolcha Health Center.

**Table 4:** Overall quality of TB care and treatment at public-health facilities in Northeast Ethiopia from January to April 2018.

Variables	DRH (%)	Boru Meda Hospital (%)	Dessie HC (%)	Kombolcha HC (%)	Average Score (%)
Outcome quality	77.5	81.2	89.1	85.9	83.4
Process quality	80	80	80	80	80
Structure quality	55.3	52.6	55.3	52.6	53.95
Average	70.9	71.3	74.8	72.8	72.5
Overall	Good	Good	Good	Good	Good

Quality score grading: very good [90–100%], good [80–90%), marginal [70–80%), poor [60–70%), and very poor (<60%).

## Data Availability

Not applicable.
